# An Entropy-Based Knowledge Measure for Atanassov’s Intuitionistic Fuzzy Sets and Its Application to Multiple Attribute Decision Making

**DOI:** 10.3390/e20120981

**Published:** 2018-12-17

**Authors:** Gang Wang, Jie Zhang, Yafei Song, Qiang Li

**Affiliations:** 1 Air and Missile Defense College, Air Force Engineering University, Xi’an 710051, China; 2 Aviation Maintenance NCO Academy, Air Force Engineering University, Xinyang 464000, China; liqiang_xy@kgd.mail.edu.cn or

**Keywords:** Atanassov’s intuitionistic fuzzy set, knowledge measure, entropy, multiple attribute decision-making

## Abstract

As the complementary concept of intuitionistic fuzzy entropy, the knowledge measure of Atanassov’s intuitionistic fuzzy sets (AIFSs) has attracted more attention and is still an open topic. The amount of knowledge is important to evaluate intuitionistic fuzzy information. An entropy-based knowledge measure for AIFSs is defined in this paper to quantify the knowledge amount conveyed by AIFSs. An intuitive analysis on the properties of the knowledge amount in AIFSs is put forward to facilitate the introduction of axiomatic definition of the knowledge measure. Then we propose a new knowledge measure based on the entropy-based divergence measure with respect for the difference between the membership degree, the non-membership degree, and the hesitancy degree. The properties of the new knowledge measure are investigated in a mathematical viewpoint. Several examples are applied to illustrate the performance of the new knowledge measure. Comparison with several existing entropy and knowledge measures indicates that the proposed knowledge has a greater ability in discriminating different AIFSs and it is robust in quantifying the knowledge amount of different AIFSs. Lastly, the new knowledge measure is applied to the problem of multiple attribute decision making (MADM) in an intuitionistic fuzzy environment. Two models are presented to determine attribute weights in the cases that information on attribute weights is partially known and completely unknown. After obtaining attribute weights, we develop a new method to solve intuitionistic fuzzy MADM problems. An example is employed to show the effectiveness of the new MADM method.

## 1. Introduction

The concept of the fuzzy set [1] was developed by Zadeh to model and process uncertain information in a much better way. By assigning the membership degree between 0 and 1 to elements with respect to a set, the fuzzy set can describe the state between “belong to” and “not belong to.” Therefore, many kinds of uncertainties that cannot be depicted by classical sets can be well-described by fuzzy sets. Since its inception, fuzzy set theory has been applied in many areas such as automatic control, pattern recognition, decision making, and more [2,3,4,5,6]. To enhance its ability and agility in handling with uncertainty, many researchers have been dedicated to extend the concept of the fuzzy set. Atanassov’s intuitionistic fuzzy set (AIFS) [7], as an extension of the fuzzy set, was defined by introducing a hesitancy degree to quantify the gap between 1 and the sum of membership degree and non-membership degree. The introduction of the hesitancy degree can depict the uncertainty on membership and non-membership grades, which bring capability of dealing with uncertainty in practical applications. Due to its advantage in coping with uncertainty, the AIFS theory has attracted attention from researchers [8,9]. Current research on the AIFS theory mainly focuses on its mathematical characteristics [10,11,12], its application in decision-making [13,14,15,16], and its relation with other uncertainty theories [17,18], etc. 

The concept of entropy was first introduced in thermodynamics. The entropy proposed by Boltzmann is a classical entropy, which is also known as Boltzmann entropy [19]. However, there is no effective method for calculating Boltzmann entropy until Shannon developed an alternative way [20]. Recently, some methods for computing Boltzmann entropy have been developed based on specific applications [19,21,22,23]. The entropy of a fuzzy set was first proposed by Zadeh [1] to depict the fuzziness. Following Zadeh’s work, De Luca and Termini [24] proposed a probabilistic entropy measure for fuzzy sets. They also put forward some axiomatic properties for the fuzzy entropy measure, according to which fuzzy entropy can be defined. Yager [25] proposed an entropy measure for fuzzy sets based on the distance between a fuzzy set and its complementation. Yager’s concept was extended by Higashi and Klir [26] to a more general kind of fuzzy complementation. Because of its importance in depicting a fuzzy set, the entropy measure of fuzzy sets has been developing to an active topic in fuzzy set theory.

Similarly, the entropy measure of AIFSs has also attracted researchers. Burillo and Bustince [27] first presented the entropy measure for AIFSs to quantify the intuitionism of AIFSs. Then, Szmidt and Kacprzyk [28] developed the axioms proposed by Burillo and Bustince [27] and introduced a new entropy measure for AIFSs based on the ratio between the nearer distance and the further distance. Hung and Yang [29] presented axiomatic definitions for entropy of AIFSs from a probabilistic point of view. It was pointed by Vlachos and Sergiadis [30] that entropy of an AIFS should capture both fuzziness and intuitionism of AIFS. Based on such a perspective, Vlachos and Sergiadis [30] defined an entropy measure based on the concept of discrimination information and cross entropy between AIFSs. Szmidt et al. [31,32] also insisted that we cannot measure the uncertainty hidden in AIFSs merely by using the entropy measure.

Knowledge measure is usually regarded as the dual measure of entropy. In fact, if an entropy cannot capture all uncertainty in AIFSs, it is not reasonable to treat knowledge and entropy as a dual measure. We hold the perspective that the uncertainty of an AIFS includes both fuzziness and intuitionism. Therefore, the entropy of an AIFS is the combination of fuzzy entropy and intuitionistic entropy. In such a case, the entropy of an AIFS can be called as intuitionistic fuzzy entropy, which captures all kinds of uncertainty in an AIFS. This kind of intuitionistic fuzzy entropy is also an uncertain measure of AIFS. Thus, the knowledge measure can be regarded as the dual measure of intuitionistic fuzzy entropy or uncertainty. Many attempts were made [32] to cope with the knowledge measure of AIFSs by combining entropy measure and the hesitancy margin. Nguyen [33] presented a knowledge measure for AIFSs based on the distance between an AIFS and the most uncertain one. Guo [34] also proposed an axiomatic definition for the knowledge measure of AIFSs and developed a new knowledge measure following his axioms. However, we find that Nguyen’s knowledge measure and Guo’s knowledge measure may bring unreasonable results due to the distance measure it used. Therefore, it is desirable to develop a new knowledge measure for AIFSs. 

To provide a more effective and reasonable knowledge measure for AIFSs, we will cope with the definition of knowledge measure of AIFSs. In this paper, we will define a new knowledge measure based on the two factors affecting knowledge amount. Our aim is to provide a new technique to measure the knowledge amount conveyed by AIFSs. Based on the intuitive analysis on the properties of knowledge amount, we present a new axiomatic definition of the knowledge measure, which differs from existing axiomatic definitions in the desired monotonicity. Afterward, a new knowledge measure is put forward together with its properties and related proofs. Comparison with other measures is made to illustrate the performance of the new knowledge measure. To validate the applicability of the new knowledge measure, we apply it in the application of multiple attribute decision making (MADM) problems in an intuitionistic fuzzy environment. A new method for solving MADM problems under the intuitionistic fuzzy condition is developed based on the new knowledge measure. An illustrative example is presented to show the effectiveness and rationality of the provided method for solving intuitionistic fuzzy MADM problems. The main contribution of this paper lies in the introduction of the effective knowledge measure, which is proved to be robust in distinguishing the knowledge amount conveyed by different AIFSs.

The rest of this paper is arranged according to the following. A brief introduction on AIFSs is proposed in the second section. A new knowledge measure for AIFSs is introduced in the third section, following the proposition axiomatic definition of knowledge measure. The properties of the new knowledge measure are also investigated in Section 3. Numerical examples and comparative analysis are presented in the fourth section to validate the performance of the new knowledge measure. The new knowledge measure is applied to MADM problems in an intuitionistic fuzzy environment in the fifth section where two models for determining attribute weights and a method for solving MADM problems are developed. In Section 5, we also use an example on the MADM problem to verify the effectiveness of the proposed method for solving MADM problems under an intuitionistic fuzzy condition. Some conclusions are presented in the last section.

## 2. Atanassov’s Intuitionistic Fuzzy Sets

The concept of the fuzzy set was introduced by Zadeh in 1965. It was defined based on the following.

**Definition** **1** **[1].**  *Let* $X=\{x_{1},x_{2},\cdots ,x_{n}\}$ *be the universe of discourse, then a fuzzy set* A *defined in* X *can be expressed as follows:*

(1) $$A=\left\{\langle x,\mu_{A}(x)\rangle |x\in X\right\}$$

*where* $\mu_{A}(x):X\to [0,1]$ *is the degree of membership for x with respect to A.*

**Definition** **2** **[7].** *An intuitionistic fuzzy set* A *in* X *defined by Atanassov can be written as:*

(2) $$A=\left\{\langle x,\mu_{A}(x),v_{A}(x)\rangle |x\in X\right\}$$

*where* $\mu_{A}(x):X\to [0,1]$ *is named as the membership degree and* $v_{A}(x):X\to [0,1]$ *is named as the non-membership degree. The sum of them is less than 1, i.e.,*

(3) $$0\leq \mu_{A}(x)+v_{A}(x)\leq 1$$

*The hesitancy degree of the element* x∈X *to the set* A *can be written as:*

(4) $$\pi_{A}(x)=1-\mu_{A}(x)-v_{A}(x)$$

*It is obvious that*$\pi_{A}(x)\in [0,1]$*,*∀x∈X.

*When* $\pi_{A}(x)=0$ *,* ∀x∈X *, the AIFS degenerates into an ordinary fuzzy set.*

*For clarity, the couple* $\langle \mu_{A}(x),v_{A}(x)\rangle$ *is usually written as* 〈μ,v〉 *, which is also called an intuitionistic fuzzy value (IFV).*

**Definition** **3** **[7].**  *For two AIFSs A and B defined in X, the following relations can be defined:*

(R1) A⊆B⇔∀x∈X$\mu_{A}(x)\leq \mu_{B}(x),v_{A}(x)\geq v_{B}(x)$;
(R2) A=B⇔∀x∈X$\mu_{A}(x)=\mu_{B}(x),v_{A}(x)=v_{B}(x)$;
(R3) $A^{C}=\left\{\langle x,v_{A}(x),\mu_{A}(x)\rangle |x\in X\right\}$, where $A^{C}$ is the complement of A.

**Definition** **4** **[7].**  *For two IFVs,* $a=\langle \mu_{a},v_{a}\rangle$ *,* $b=\langle \mu_{b},v_{b}\rangle$ *, the partial order between them is defined as:* $a\leq b\Leftrightarrow \mu_{a}\leq \mu_{b},v_{a}\geq v_{b}$ *.*

*For a linear order of IFVs, Chen and Tan [35] defined the score function of IFV as* $S(a)=\mu_{a}-v_{a}$ *to rank multiple IFVs. Then Hong and Choi [36] defined an accuracy function* $H(a)=\mu_{a}+v_{a}$ *to measure the accuracy of an IFV. Based on score and accuracy functions, Xu and Yager [37] developed a linear order relation for IFVs. Given two IFVs* $a=\langle \mu_{a},v_{a}\rangle$ *and* $b=\langle \mu_{b},v_{b}\rangle$ *, we have the following relations:* S(a)>S(b)⇒a>b *;* S(a)=S(b),H(a)=H(b)⇒a=b *;* S(a)=S(b),H(a)>H(b)⇒a>b *.*

## 3. A New Knowledge Measure for AIFSs

Let $X=\{x_{1},x_{2},\cdots ,x_{n}\}$ be the discourse universe. For an AIFS *A* defined in *X*, the measure used to quantify its knowledge amount should have some intuitive properties. Rationally, the knowledge measure must be a nonnegative function. The knowledge amount of an AIFS and that of its complement should be equal to each other. When an AIFS degrade into a classical fuzzy set, its knowledge amount increases with the difference between the membership degree and the non-membership degree. Moreover, for an AIFS in which the difference between membership and non-membership grades are fixed, the knowledge amount behaves dually to the hesitancy degree. Since a crisp set provided the maximum knowledge, the knowledge amount reaches its maximum if the AIFS reduces into a crisp set. On the contrary, $\pi_{A}(x_{i})=1$ for each i=1,2,⋯,n indicates full ignorance, so the knowledge amount reaches its minimum value 0 in such a case. Moreover, in the condition of $\mu_{A}(x_{i})=v_{A}(x_{i})\neq 0$, the greater hesitant degree $\pi_{A}(x_{i})$ implies the less uncertainty amount.

Having these intuitive properties in mind, we can propose the following axiomatic definition for the knowledge measure of AIFSs.

**Definition** **5.**  *For an AIFS A defined in* $X=\{x_{1},x_{2},\cdots ,x_{n}\}$ *, its knowledge measure is a mapping* K:AIFS→[0,1] *satisfying the following properties:*

*(KP1)*  K(A)=1 *if and only if A is a crisp set.*
*(KP2)*  K(A)=0*if and only if*$\pi_{A}(x_{i})=1$*,*∀i∈{1,2,⋯,n}.
*(KP3)*  K(A) *is increasing with* $\Delta_{A}(x_{i})=\left|\mu_{A}(x_{i})-v_{A}(x_{i})\right|$ *and decreasing with* $\pi_{A}(x_{i})$ *,* i=1,2,⋯,n *.*
*(KP4)*  $K(A^{C})=K(A)$ *.*

We note that in References [33,34], the third property of entropy for AIFS is stated as: K(A)≥K(B) if A is less fuzzy than B, i.e., A⊆B for $\mu_{B}(x)\leq v_{B}(x)$, ∀x∈X, or A⊇B for $\mu_{B}(x_{i})\geq v_{B}(x_{i})$, ∀x∈X. We can see that this property indicates that the knowledge amount behaves dually to the fuzziness of an AIFS, which does not consider the impact of the hesitancy degree. The condition A⊆B and $\mu_{B}(x_{i})\leq v_{B}(x_{i})$ indicate $\mu_{A}(x_{i})\leq \mu_{B}(x_{i})\leq v_{B}(x_{i})\leq v_{A}(x_{i})$. Therefore, $\left|\mu_{A}(x_{i})-v_{A}(x_{i})\right|\geq \left|\mu_{B}(x_{i})-v_{B}(x_{i})\right|$. Similarly, given A⊇B and $\mu_{B}(x_{i})\geq v_{B}(x_{i})$, we have $\mu_{A}(x_{i})\geq \mu_{B}(x_{i})\geq v_{B}(x_{i})\geq v_{A}(x_{i})$, $\left|\mu_{A}(x_{i})-v_{A}(x_{i})\right|\geq \left|\mu_{B}(x_{i})-v_{B}(x_{i})\right|$. Therefore, we can say the knowledge measure is decreasing with fuzziness. It may be arbitrary to give the property that K(A)≥K(B) if A is less fuzzy than B. Hence, when the hesitancy degree is fixed, we also can say K(A)≥K(B) if A is less fuzzy than B, which is consistent with our proposed property. Moreover, the cases of $\mu_{A}(x_{i})\leq \mu_{B}(x_{i})\leq v_{B}(x_{i})\leq v_{A}(x_{i})$ and $\mu_{A}(x_{i})\geq \mu_{B}(x_{i})\geq v_{B}(x_{i})\geq v_{A}(x_{i})$ indicate that $\left|\mu_{A}(x_{i})-v_{A}(x_{i})\right|\geq \left|\mu_{B}(x_{i})-v_{B}(x_{i})\right|$, but $\left|\mu_{A}(x_{i})-v_{A}(x_{i})\right|\geq \left|\mu_{B}(x_{i})-v_{B}(x_{i})\right|$ cannot infer $\mu_{A}(x_{i})\leq \mu_{B}(x_{i})\leq v_{B}(x_{i})\leq v_{A}(x_{i})$ or $\mu_{A}(x_{i})\geq \mu_{B}(x_{i})\geq v_{B}(x_{i})\geq v_{A}(x_{i})$. Generally, it has been known that the fuzziness of an AIFS including classical fuzzy sets is related to the difference between membership and non-membership grades. Thus, it is not comprehensive to equate the concept of fuzziness to the relation of inclusion.

The above analysis indicates that the amount of knowledge for AIFSs is decreasing with fuzziness, but there is no determinative relation between them if the hesitancy degree is ignored. The third property in Reference [34] is a strict constraint for defining the knowledge measure. Similarly, this kind of properties for intuitionistic fuzzy entropy and uncertainty measures [28,33,38] are also incomplete and stricter because of the absence of the hesitancy degree and the equivalence between fuzziness and the inclusion relation of AIFSs.

It is known that several divergence measures have been proposed based on Shannon entropy. Kullback–Leibler divergence (K–L divergence) [39] is one of the most popular divergence measures developed from Shannon entropy [20]

Let *X* be a discrete random variable. *P*_1_ and *P*_2_ are two probability distributions for *X*. The K–L divergence between *P*_1_ and *P*_2_ is defined by the equation below [35].

(5) $$D_{KL}(P_{1},P_{2})=\frac{1}{\ln2}\sum_{j}p_{1j}\ln\frac{p_{1j}}{p_{2j}}$$

where *p_ij_* is the probability of occurrence of the value *X* = *x_j_* for each of the probability distribution *P_i_*, *I* = 1, 2. To construct a symmetric divergence measure, the divergence measure between *P*_1_ and *P*_2_ can be defined by the formula below.

(6) $$D_{KL}^{S}(P_{1},P_{2})=D_{KL}(P_{1},P_{2})+D_{KL}(P_{2},P_{1})=\frac{1}{\ln2}\sum_{j}(p_{1j}-p_{2j})\ln\frac{p_{1j}}{p_{2j}}$$

In applications, to avoid the undefined case of zero denominator, the symmetric divergence measure can be modified by the equation below.

(7) $$D_{KL}^{SM}(P_{1},P_{2})=\frac{1}{\ln2}\sum_{j}(p_{1j}-p_{2j})\ln\frac{1+p_{1j}}{1+p_{2j}}$$

Then, we can construct a knowledge measure for an AIFS *A* defined in $X=\{x_{1},x_{2},\cdots ,x_{n}\}$ by measuring the divergence between *A* and the most uncertain AIFS U={〈x,0,0〉|x∈X}. Based on the previously mentioned axiomatic definition for the knowledge measure of AIFSs and the modified divergence measure in Reference (7), the knowledge measure can be expressed by the following equation.

(8) $$K_{S}(A)=\frac{1}{2n\ln2}\sum_{i=1}^{n}\left[\Delta_{A}(x_{i})\ln\left(\Delta_{A}(x_{i})+1\right)+\left(\pi_{A}(x_{i})-1\right)\ln\frac{\pi_{A}(x_{i})+1}{2}\right]$$

where $\Delta_{A}(x_{i})=\left|\mu_{A}(x_{i})-v_{A}(x_{i})\right|$, i=1,2,⋯,n.

Then, we will prove that the proposed knowledge measure *K_S_*(*A*) satisfies all properties in Definition 5.

**Theorem** **1.** *For an AIFS A defined in* $X=\{x_{1},x_{2},\cdots ,x_{n}\}$ *, then* $K_{S}(A)=1$ *if and only if A is a crisp set.*

**Proof.**  (1) For a crisp subset of *X*, we have $\pi_{A}(x)=0$, and $\Delta_{A}(x)=1$, ∀x∈X. Then we can get $K_{S}(A)=1$. For AIFS A defined in $X=\{x_{1},x_{2},\cdots ,x_{n}\}$, we can get $\Delta_{A}(x_{i})=\left|\mu_{A}(x_{i})-v_{A}(x_{i})\right|\in [0,1]$ and $\Delta_{A}(x_{i})\in [0,1]$. Then, it follows that $1\leq \Delta_{A}(x)+1\leq 2$, $1/2\leq \left(1+\Delta_{A}(x)\right)/2\leq 1$ and $-1\leq \pi_{A}(x)-1\leq 0$.

Considering the function f(x,y)=xln(x+1)+(y−1)ln(0.5(y+1)) with x,y∈[0,1], we have:

$\frac{\partial f(x,y)}{\partial x}=\ln(x+1)+\frac{x}{x+1}>0$, $\frac{\partial f(x,y)}{\partial y}=\ln\frac{y+1}{2}+\frac{y-1}{y+1}<0$.

This indicates that *f*(*x*,*y*) is strictly increasing with *x* and strictly decreasing with *y.* Hence, *f*(*x*,*y*) has a single maximum point (1,0) where xln(x+1)=ln2, (y−1)ln(0.5(y+1))=ln2 and f(x,y)=2ln2.

Therefore, each part of $K_{S}(A)$, i.e., $\Delta_{A}(x_{i})\ln\left(\Delta_{A}(x_{i})+1\right)$ and $\left(\pi_{A}(x_{i})-1\right)\ln\frac{\pi_{A}(x_{i})+1}{2}$, is less than ln2. By $K_{S}(A)=1$, we can infer that $\Delta_{A}(x_{i})\ln\left(\Delta_{A}(x_{i})+1\right)=\ln2$ and $\left(\pi_{A}(x_{i})-1\right)\ln\frac{\pi_{A}(x_{i})+1}{2}=\ln2$ for all $x_{i}\in X$, which indicates that $\pi_{A}(x_{i})=0$, and $\Delta_{A}(x_{i})=1$ for all $x_{i}\in X$.

Therefore, *A* is a crisp set.

Therefore, we have $K_{S}(A)=1$ if and only if *A* is a crisp set. □

**Theorem** **2.** *For an AIFS A defined in* $X=\{x_{1},x_{2},\cdots ,x_{n}\}$ *, then* $K_{S}(A)=0$ *if and only if* $\pi_{A}(x_{i})=1$ *for each* $x_{i}\in X$ *.*

**Proof.**  The condition $\pi_{A}(x_{i})=1$ $\forall x_{i}\in X$ indicates that $\mu_{A}(x_{i})=v_{A}(x_{i})=0$ $\forall x_{i}\in X$. Therefore, we have $\Delta_{A}(x_{i})=0$, $\forall x_{i}\in X$. Then $K_{S}(A)=0$ can be yielded.

For $x_{i}\in X$, given $1\leq \Delta_{A}(x_{i})+1\leq 2$, $1/2\leq \left(1+\Delta_{A}(x_{i})\right)/2\leq 1$, and $-1\leq \pi_{A}(x_{i})-1\leq 0$, we can get $\Delta_{A}(x_{i})\ln\left(\Delta_{A}(x_{i})+1\right)\geq 0$ and $\left(\pi_{A}(x_{i})-1\right)\ln\frac{\pi_{A}(x_{i})+1}{2}\geq 0$. $K_{S}(A)=0$ indicates that $\Delta_{A}(x_{i})\ln\left(\Delta_{A}(x_{i})+1\right)=0$ and $\left(\pi_{A}(x_{i})-1\right)\ln\frac{\pi_{A}(x_{i})+1}{2}=0$ for all $x_{i}\in X$. Therefore, we have $\pi_{A}(x_{i})=1$ for each $x_{i}\in X$.

Then, we can get that $K_{S}(A)=0$ if and only if $\pi_{A}(x_{i})=1$ for each $x_{i}\in X$. □

**Theorem** **3.** *For an AIFS A defined in* $X=\{x_{1},x_{2},\cdots ,x_{n}\}$ *,* $K_{S}(A)$ *is increasing with* $\Delta_{A}(x_{i})=\left|\mu_{A}(x_{i})-v_{A}(x_{i})\right|$ *and decreasing with* $\pi_{A}(x_{i})$ *,* i=1,2,⋯,n *.*

**Proof.**  In the proof of Theorem 1, it has been pointed out that the function f(x,y)=xln(x+1)+(y−1)ln(0.5(y+1)) with x,y∈[0,1] is strictly increasing with *x* and strictly decreasing with *y*.

Therefore, the summation of all $f(\Delta_{A}(x_{i}),\pi_{A}(x_{i}))$ is increasing with $\Delta_{A}(x_{i})$ and decreasing with $\pi_{A}(x_{i})$.

Then, it follows that $K_{S}(A)$ is increasing with $\Delta_{A}(x_{i})=\left|\mu_{A}(x_{i})-v_{A}(x_{i})\right|$ and decreasing with $\pi_{A}(x_{i})$, i=1,2,⋯,n. □

**Theorem** **4.** *For an AIFS, A defined in* $X=\{x_{1},x_{2},\cdots ,x_{n}\}$ *,* $K_{S}(A^{C})=K_{S}(A)$ *.*

**Proof.**  This is straightforward by the definition of $A^{C}$ and $K_{S}$. □

Theorem 1–4 illustrates that the proposed mapping $K_{S}:AIFS\to [0,1]$ satisfies all properties in the axiomatic definition of the knowledge measure. Therefore, $K_{S}$ is a knowledge measure of AIFSs.

**Figure 1:**
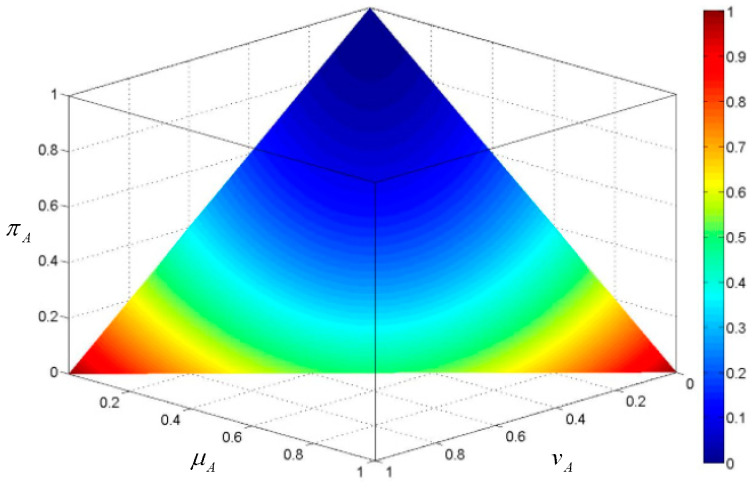
The value of the knowledge measure KS (A).

To provide a visual perception on the proposed knowledge measure, we consider an AIFS *A* defined in *X* = {*x*}. Based on the geometric interpretation of AIFSs proposed by Szmidt and Kacprzyk [28], the values of the knowledge amount are projected to the hyper plane in the unit intuitionistic fuzzy cube, as shown in Figure 1. The change of knowledge amount according to the distribution of membership and non-membership grades can be reflected by this figure. We note that, in the condition of $\mu_{A}=v_{A}$, the knowledge amount is decreasing with the hesitancy degree. For a fixed hesitancy degree, the knowledge amount is increasing the difference between the membership degree and the non-membership degree. This is consistent with the proposed axiomatic properties of the knowledge measure.

## 4. Numerical Examples

In this section, the performance of the proposed knowledge measure *K_S_* will be validated based on two numerical examples. To illustrate the effectiveness and performance of the proposed Biparametric uncertainty measure for AIFSs, some existing intuitionistic fuzzy entropy measures will be adopted for comparison. Therefore, we first recall some widely used entropy measures for AIFSs.

The entropy measure proposed by Zeng and Li [40] is shown below.

(9) $$E_{ZL}(A)=1-\frac{1}{n}\sum_{i=1}^{n}\left|\mu_{A}(x_{i})-v_{A}(x_{i})\right|$$

The entropy measure proposed by Burillo and Bustince [27] is shown below.

(10) $$E_{BB}(A)=\frac{1}{n}\sum_{i=1}^{n}\left(1-\mu_{A}(x_{i})-v_{A}(x_{i})\right)$$

The entropy measure proposed by Szmidt and Kacprzyk [28] is based on the equation below.

(11) $$E_{SK}(A)=\frac{1}{n}\sum_{i=1}^{n}\frac{\min\left(\mu_{A}(x_{i}),v_{A}(x_{i})\right)+\pi_{A}(x_{i})}{\max\left(\mu_{A}(x_{i}),v_{A}(x_{i})\right)+\pi_{A}(x_{i})}$$

The entropy measure proposed by Vlachos and Sergiadis [30] is shown below.

(12) $$E_{VS}(A)=-\frac{1}{n\ln2}\sum_{i=1}^{n}\left(\mu_{A}(x_{i})\ln\mu_{A}(x_{i})+v_{A}(x_{i})\ln v_{A}(x_{i})-\left(1-\pi_{A}(x_{i})\right)\ln\left(1-\pi_{A}(x_{i})\right)\right)+\frac{1}{n}\sum_{i=1}^{n}\pi_{A}(x_{i})$$

The entropy measure proposed by Hung and Yang [29] is illustrated below.

(13) $$E_{HC}^{2}(A)=\frac{1}{n}\sum_{i=1}^{n}\left(1-{\left(\mu_{A}(x_{i})\right)}^{2}-{\left(v_{A}(x_{i})\right)}^{2}-{\left(\pi_{A}(x_{i})\right)}^{2}\right)$$

The knowledge measure proposed by Szmidt, Kacprzyk, and Bujnowski [32] is shown in the equation below.

(14) $$K_{SKB}(A)=1-\frac{1}{2n}\left[\sum_{i=1}^{n}\frac{\min\left(\mu_{A}(x_{i}),v_{A}(x_{i})\right)+\pi_{A}(x_{i})}{\max\left(\mu_{A}(x_{i}),v_{A}(x_{i})\right)+\pi_{A}(x_{i})}+\pi_{A}(x_{i})\right]$$

Nguyen’s knowledge measure [33] equals the following.

(15) $$K_{N}(A)=\frac{1}{n\sqrt{2}}\sum_{i=1}^{n}\sqrt{{\left(\mu_{A}(x_{i})\right)}^{2}+{\left(v_{A}(x_{i})\right)}^{2}+{\left(\mu_{A}(x_{i})+v_{A}(x_{i})\right)}^{2}}$$

Guo’s knowledge measure [34] is based on the equation below.

(16) $$K_{G}(A)=1-\frac{1}{2n}\sum_{i=1}^{n}\left(1-\left|\mu_{A}(x_{i})-v_{A}(x_{i})\right|\right)\left(1+\pi_{A}(x_{i})\right)$$

**Example** **1.** *Four AIFSs defined in* X *= {x} are given as: A*_1_ *= <x,0.5,0.5>, A*_2_ *= <x,0.25,0.25>, A*_3_ *= <x,0.25,0.5>, A*_4_ *= <x, 0.2,0.3>.*

According to the eight existing measures and our proposed uncertainty measure, we can calculate the uncertainty degree and amount of knowledge. The comparative results are shown in Table 1.

From Table 1, we can see that the entropy measure *E_ZL_*, *E_SK_*, and *E_VS_* cannot discriminate the uncertainty of *A*_1_ and *A*_2_. Since the entropy measure *E_BB_* is defined based on the hesitancy degree of AIFSs, it assigns zero uncertainty to *A*_1_, which is unreasonable. Moreover, the uncertainty grades of *A*_2_ and *A*_4_ cannot be distinguished by *E_BB_* because their hesitancy degrees are identical. It is also shown that uncertainty grades of *A*_2_ and *A*_4_ calculated by the entropy measure *E*^2^*_HC_* are equal to each other.

It is also shown that the knowledge measures *K_SKB_*, *K_N_*, *K_G_*, together with our proposed measure *K_S_* can discriminate these four AIFSs well from the perspective of the amount of knowledge. For the ranking order of the knowledge amount, we see that the measures *K_SKB_* and *K_G_* yield the results *K*(*A*_3_) > *K*(*A*_1_) > *K*(*A*_4_) > *K*(*A*_2_). When the knowledge measure *K_N_* is applied, we can obtain that *K*(*A*_1_) > *K*(*A*_3_) > *K*(*A*_2_) > *K*(*A*_4_). Our proposed measure *K_S_* leads to the order *K*(*A*_1_) > *K*(*A*_3_) > *K*(*A*_4_) > *K*(*A*_2_). Comparing AIFSs *A*_1_ and *A*_3_, we can see that the difference between the membership and non-membership degree of *A*_1_ is less than that of *A*_3_. However, *A*_3_ has more of a hesitancy degree than *A*_1_. According to the monotonicity of the knowledge measure proposed in Definition 5, the knowledge amount of *A*_1_ and *A*_3_ cannot be determined. But for *A*_2_ and *A*_4_, it is shown that *A*_2_ has less Δ, i.e., the difference between its membership and non-membership grades, and more hesitancy degree π than *A*_4_. Therefore, *A*_4_ should convey a higher amount of knowledge than *A*_2_. In such a case, the knowledge measure *K_N_* is less reasonable than the other three knowledge measures.

This example indicates that our proposed knowledge measure is effective in measuring the knowledge amount. It is competent to reflect the intuitive relation between different AIFSs.

**Example** **2.** *Nine AIFSs defined in* X *= {x} are considered. These AIFSs are given as: A*_1_ *= <x,0.7,0.2>, A*_2_ *= <x,0.5,0.3>, A*_3_ *= <x,0.5,0>, A*_4_ *= <x,0.5,0.5>, A*_5_ *= <x,0.5,0.4>, A*_6_ *= <x,0.6,0.2>, A*_7_ *= <x,0.4,0.4>, A*_8_ *= <x,1,0>, and A*_9_ *= <x,0,0>.*

Based on five widely used entropy measures *E_ZL_*, *E_BB_*, *E_SK_*, *E_VS_*, and *E*^2^*_HC_*, three knowledge measures *K_SKB_*, *K_N_*, and *K_G_*, and our proposed measures *K_S_*, we can calculate the uncertainty of these nine AIFSs. The results are shown in Table 2.

We note that the AIFS *A*_9_ = <*x*,0,0> is the most uncertain one with the least amount of knowledge. Therefore, its uncertainty measure should be 1 and the knowledge amount is 0. It can be seen that only *E*^2^*_HC_* cannot produce reasonable results for AIFS *A*_9_ = <*x*,0,0>. For AIFS *A*_8_ = <*x*,1,0>, it is the most certain one conveying the maximum knowledge amount. Hence, its uncertainty degree is 0 and the knowledge amount is 1 since the results were produced by all measures in Table 2. Moreover, we can see that all entropy measures may bring counter-intuitive results, which have been highlighted in a bold type in Table 2. These unreasonable results show that these entropy measures are not competent to distinguishing different AIFSs. The knowledge measure *K_SKB_* assigned the same knowledge amount to AIFSs *A*_3_ = <*x*,0.5,0> and *A*_4_ = <*x*,0.5,0.5>, which indicates the poorer discriminant ability of *K_SKB_*. By the proposed axiomatic definition, we cannot rank the knowledge amount of *A*_3_ and *A*_4_ since $\Delta_{A_{4}}$ and $\pi_{A_{4}}$ is greater that $\Delta_{A_{5}}$ and $\pi_{A_{5}}$, respectively. Comparing *A*_1_ and *A*_3_, we find that $\Delta_{A_{1}}=\Delta_{A_{3}}$ and $\pi_{A_{1}}<\pi_{A_{3}}$. Therefore, *A*_1_ has a greater knowledge amount than *A*_3_, which can be yielded by all knowledge measures. For AIFSs *A*_1_ and *A*_5_, they have the same hesitancy degree. The greater difference between the membership and non-membership grades of *A*_1_ brings more knowledge amount than *A*_5_, as shown by the results of all knowledge measures. We can also rank the knowledge amount of AIFSs *A*_2_, *A*_6_, and *A*_7_ in the same way. We note that AIFSs *A*_4_, *A*_7_, and *A*_9_ have the same Δ, so the less hesitancy degree indicates a greater knowledge amount. Thus, the knowledge conveyed by *A*_4_, *A*_7_, and *A*_9_ should be ranked as *K*(*A*_4_) > *K*(*A*_7_) > *K*(*A*_9_). It is shown that all knowledge measures can provide this ranking order. This example tells us that our proposed knowledge measure is effective to distinguish the knowledge amount of different AIFSs.

**Example** **3.** *We consider an AIFS* A *defined in* X *= {6,7,8,9,10}. The AIFS A is defined as:*

A={〈6,0.1,0.8〉,〈7,0.3,0.5〉,〈8,0.5,0.5〉,〈9,0.9,0〉,〈10,1,0〉}.

De et al. [41] have defined an exponent operations for AIFS A defined in X. Given a non-negative real number *m*, $A^{m}$ is defined as:

(17) $$A^{m}=\left\{\langle x,{\left(\mu_{A}(x)\right)}^{m},1-{\left(1-v_{A}(x)\right)}^{m}\rangle x\in X|\right\}$$

Based on the operations in Equation (17), we have:

$A^{0.5}=\left\{\langle 6,0.316,0.553\rangle ,\langle 7,0.548,0.293\rangle ,\langle 8,0.707,0.293\rangle ,\langle 9,0.949,0\rangle ,\langle 10,1,0\rangle \right\}$.

$A^{2}=\left\{\langle 6,0.010,0.960\rangle ,\langle 7,0.090,0.750\rangle ,\langle 8,0.250,0.750\rangle ,\langle 9,0.810,0\rangle ,\langle 10,1,0\rangle \right\}$.

$A^{3}=\left\{\langle 6,0.001,0.992\rangle ,\langle 7,0.027,0.875\rangle ,\langle 8,0.125,0.875\rangle ,\langle 9,0.729,0\rangle ,\langle 10,1,0\rangle \right\}$.

$A^{4}=\left\{\langle 6,0.0001,0.998\rangle ,\langle 7,0.008,0.938\rangle ,\langle 8,0.062,0.938\rangle ,\langle 9,0.656,0\rangle ,\langle 10,1,0\rangle \right\}$.

Considering the characterization analysis on linguistic variables, we can regard the AIFS *A* as ‘‘LARGE’’ in *X*. Correspondingly, AIFSs *A*^0.5^, *A*^2^, *A*^3^ and *A*^4^ can be regarded as “More or less LARGE”, “Very LARGE”, “Quite very LARGE”, and “Very very LARGE”, respectively.

Intuitively, from *A*^0.5^ to *A*^4^, the uncertainty hidden in them becomes less and the knowledge amount conveyed by them increases. So the following relations hold:

(18) $$E\left(A^{0.5}\right)>E\left(A\right)>E\left(A^{2}\right)>E\left(A^{3}\right)>E\left(A^{4}\right)$$

(19) $$K\left(A^{0.5}\right)<K\left(A\right)<K\left(A^{2}\right)<K\left(A^{3}\right)<K\left(A^{4}\right)$$

To make a comparison, entropy measures *E_ZL_*, *E_BB_*, *E_VS_*, *E*^2^*_HC_*, and knowledge measures *K_SKB_*, *K_N_*, and *K_G_* are employed to facilitate analysis. In Table 3, we present the results obtained based on different measures to facilitate comparative analysis.

We can note that the AIFS *A* will be assigned more entropy than the AIFS *A*^0.5^ when entropy measures *E_ZL_*, *E_BB_*, and *E_SK_* are applied. The ranking orders obtained based on these measures are listed below.

$E_{ZL}(A)>E_{ZL}(A^{0.5})>E_{ZL}(A^{2})>E_{ZL}(A^{3})>E_{ZL}(A^{4})$.

$E_{BB}(A)>E_{BB}(A^{0.5})>E_{BB}(A^{2})>E_{BB}(A^{3})>E_{BB}(A^{4})$.

$E_{SK}(A)>E_{SK}(A^{0.5})>E_{SK}(A^{2})>E_{SK}(A^{3})>E_{SK}(A^{4})$.

It is shown that these ranked orders do not satisfy intuitive analysis in Equation (18), while other entropy measures can induce desirable results. In this example, *E*^2^*_HC_* and *E_VS_* perform well. This illustrates that these entropy measures are not robust enough to distinguish the uncertainty of AIFSs with linguistic information.

Moreover, the results produced by knowledge measures *K_SKB_*, *K_N_*, and *K_G_* are also not reasonable, which are shown as the equations below.

$K_{SKB}(A)<K_{SKB}(A^{0.5})<K_{SKB}(A^{2})<K_{SKB}(A^{3})<K_{SKB}(A^{4})$,

$K_{N}(A)<K_{N}(A^{0.5})<K_{N}(A^{2})<K_{N}(A^{3})<K_{N}(A^{4})$,

$K_{G}(A)<K_{G}(A^{0.5})<K_{G}(A^{2})<K_{G}(A^{3})<K_{G}(A^{4})$.

However, our proposed knowledge measure *K_S_* indicates that:

$K_{S}(A^{0.5})<K_{S}(A)<K_{S}(A^{2})<K_{S}(A^{3})<K_{S}(A^{4})$.

Therefore, the knowledge measures *K_SVB_*, *K_N_*, and *K_G_* are not suitable for differentiating the knowledge amount conveyed by AIFSs. The effectiveness of our proposed knowledge measure *K^I^* is indicated by this example once again.

From the above examples, we can conclude that entropy measures *E_ZL_*, *E_BB_*, *E_SK_*, *E_VS_*, and *E*^2^*_HC_* perform poor because of their lack of robustness and discriminability. Our proposed knowledge measure performs much better than knowledge measures *K_SVB_*, *K_N_*, and *K_G_*.

## 5. Application in Solving Intuitionistic Fuzzy MADM

In this section, the proposed knowledge measure will be applied in solving multiple attribute decision making (MADM) problems. The MADM problems to be considered can be described below.

All alternatives consist of a set denoted as $G=\{G_{1},G_{2},\cdots ,G_{m}\}$. The set of all considered attributes is expressed as $A=\{A_{1},A_{2},\cdots ,A_{n}\}$. The weight vector of attributes is $w={\left(w_{1},w_{2},\cdots ,w_{n}\right)}^{T}$ with $\sum_{i=1}^{n}w_{i}=1$. Due to the limitation of the decision-maker’s knowledge and expertise, an intuitionistic fuzzy form expresses the evaluation information provided under each attribute. The intuitionistic fuzzy decision matrix given by the decision maker is expressed as:

(20) $$R=\begin{array}{ll} & \begin{array}{cccc} \;A_{1} & \;A_{2} & \;\cdots & \;A_{n} \end{array}\\ \begin{array}{l} G_{1}\\ G_{2}\\ \vdots \\ G_{m} \end{array} & \left(\begin{array}{cccc} \langle \mu_{11}^{},v_{11}^{}\rangle & \langle \mu_{12}^{},v_{12}^{}\rangle & \cdots & \langle \mu_{1n}^{},v_{1n}^{}\rangle \\ \langle \mu_{21}^{},v_{21}^{}\rangle & \langle \mu_{22}^{},v_{22}^{}\rangle & \cdots & \langle \mu_{2n}^{},v_{2n}^{}\rangle \\ \vdots & \vdots & \ddots & \vdots \\ \langle \mu_{m1}^{},v_{m1}^{}\rangle & \langle \mu_{m2}^{},v_{m2}^{}\rangle & \cdots & \langle \mu_{mn}^{},v_{mn}^{}\rangle \end{array}\right) \end{array}$$

where the $r_{ij}^{}=\langle \mu_{ij}^{},v_{ij}^{}\rangle$ evaluation result of alternative *G_i_* with respect to the attribute *A*_j_, provided by the decision-maker in the form of an intuitionistic fuzzy value.

When solving MADM problems, the weights of attributes are important. If the attribute weights are completely known, this MADM problem can be solved by aggregating all intuitionistic fuzzy information under different attributes and comparing the final intuitionistic fuzzy values. However, in a practical application, the attribute weights are usually partially known or completely unknown [42]. Therefore, the attribute weights must be determined before solving the MADM problems. The attribute weights can be empirically assigned by experts. However, this method is subjective and the partial information on the attribute weights may not be used sufficiently. Therefore, we can propose a new model to determine the attribute weights based on the proposed knowledge measure. Generally, we hope the evaluation results of all alternatives under one attribute are distinguished enough to facilitate our decision making. Therefore, we can set the total knowledge amount as the objective function of optimization. By maximizing the sum of the knowledge amount under all attributes, we can construct the following models.

(21) $$\begin{array}{l} Max\;T=\sum_{j=1}^{n}w_{j}\sum_{i=1}^{m}K_{ij}\\ \;s.t.\;\left\{ \begin{array}{l} w\in H,\\ \sum_{j=1}^{n}w_{j}=1,\\ w_{j}\geq 0,j=1,2,\cdots ,n \end{array}\right. \end{array}$$

where *H* is the set of all incomplete information about attribute weights and $K_{ij}$ is the knowledge amount calculated by our proposed knowledge measure, i.e., $K_{ij}=K_{S}(r_{ij})$.

When the attribute weights are completely unknown, the attribute under which the total knowledge is greater should be assigned more weight. Therefore, we can calculate attribute weights by using the following equation.

(22) $$w_{j}=\frac{\sum_{i=1}^{m}K_{ij}}{\sum_{j=1}^{n}\sum_{i=1}^{m}K_{ij}}$$

When the attribute weights are obtained, the intuitionistic fuzzy MADM problems can be solved by aggregating the intuitionistic fuzzy information under all attributes. Many weighted aggregation operators have been proposed for integrating intuitionistic fuzzy information. In this section, we will use the well-known intuitionistic fuzzy weighted averaging (IFWA) operator [43] to solve the MADM problems under an intuitionistic fuzzy environment. The intuitionistic fuzzy information of alternative *G_i_* can be aggregated to an intuitionistic fuzzy value *z_i_*, which is expressed as:

(23) $$z_{i}=\langle \mu_{i},v_{i}\rangle =IFWA_{w}\left(\langle \mu_{ij}^{},v_{ij}^{}\rangle ,\langle \mu_{ij}^{},v_{ij}^{}\rangle ,\cdots ,\langle \mu_{ij}^{},v_{ij}^{}\rangle \right)=\langle 1-\prod_{j=1}^{n}{\left(1-\mu_{ij}^{}\right)}^{w_{j}},\prod_{j=1}^{n}{\left(v_{ij}^{}\right)}^{w_{j}}\rangle$$

Then the score function *S*(*z_i_*) and accuracy function *H*(*z_i_*) of *z_i_* (i=1,2,⋯,m) can be calculated as:

(24) $$S(z_{i})=\mu_{i}-v_{i}$$

(25) $$H(z_{i})=\mu_{i}+v_{i}$$

Lastly, all alternatives can be ranked into an order according to the linear order relation between IFVs based on score function and accuracy function.

Next, an example will be applied to demonstrate the performance of our proposed method for solving MADM problems in an intuitionistic fuzzy condition.

**Example** **4.** *A capital company will invest a sum of money to a project. Considering the complexity of economic development, they choose five companies as candidates. Five companies to be considered are shown as:*

*G*_1_: A cell phone company.
*G*_2_: A food company.
*G*_3_: An automobile sales company.
*G*_4_: A computer company.
*G*_5_: a TV company.

The investment company evaluates these five companies from four attributes, which are:

*A*_1_: The investment risk.
*A*_2_: The capital gain.
*A*_3_: The social and political impact.
*A*_4_: The environmental impact.

The evaluated results with intuitionistic fuzzy information are shown as:

$$R=\begin{array}{ll} & \begin{array}{cccc} \;A_{1} & \;A_{2} & \;A_{3} & \;A_{4} \end{array}\\ \begin{array}{l} G_{1}\\ G_{2}\\ G_{3}\\ G_{4}\\ G_{5} \end{array} & \left(\begin{array}{llll} \langle 0.5,0.4\rangle & \langle 0.6,0.3\rangle & \langle 0.3,0.6\rangle & \langle 0.2,0.7\rangle \\ \langle 0.7,0.3\rangle & \langle 0.7,0.2\rangle & \langle 0.7,0.2\rangle & \langle 0.4,0.5\rangle \\ \langle 0.6,0.4\rangle & \langle 0.5,0.4\rangle & \langle 0.5,0.3\rangle & \langle 0.6,0.3\rangle \\ \langle 0.8,0.1\rangle & \langle 0.6,0.3\rangle & \langle 0.3,0.4\rangle & \langle 0.2,0.6\rangle \\ \langle 0.6,0.2\rangle & \langle 0.4,0.3\rangle & \langle 0.7,0.1\rangle & \langle 0.5,0.3\rangle \end{array}\right) \end{array}$$

**Case** **1.**  *The information on the attribute weights is incomplete. The partial information on attribute weights is listed in set* H, $H=\{0.15\leq w_{1}\leq 0.2;0.16\leq w_{2}\leq 0.18;0.3\leq w_{3}\leq 0.35;0.3\leq w_{4}\leq 0.45\}$.

The total knowledge amount under each attribute can be calculated by the equations below.

$K_{1}=\sum_{i=1}^{5}K_{1j}=\sum_{i=1}^{5}K_{S}(r_{1j})=2.5663$; $K_{2}=\sum_{i=1}^{5}K_{2j}=\sum_{i=1}^{5}K_{S}(r_{2j})=2.0436$;

$K_{3}=\sum_{i=1}^{5}K_{3j}=\sum_{i=1}^{5}K_{S}(r_{3j})=2.0230$; $K_{4}=\sum_{i=1}^{5}K_{4j}=\sum_{i=1}^{5}K_{S}(r_{4j})=2.0872$.

The optimal model to determine the attribute weights can be constructed as:

$\begin{array}{l} Max\;T=2.5663w_{1}+2.0436w_{2}+2.0230w_{3}+2.0872w_{4}\\ \;s.t.\;\left\{ \begin{array}{l} w\in H\\ \sum_{j=1}^{4}w_{j}=1,\\ w_{j}\geq 0,j=1,2,\cdots ,4 \end{array}\right. \end{array}$

Then the weighting vector of the attribute can be obtained as:

$w={\left(0.20,0.16,0.30,0.34\right)}^{T}$

Using the IFWA operator, we can aggregate the intuitionistic fuzzy information for each alternative with respect to all attributes. Then we have:

*Z*_1_ = <0.3738, 0.5218>, *Z*_2_ = <0.6203, 0.2962>, *Z*_3_ = <0.5568, 0.3327>,

*Z*_4_ = <0.4787, 0.3323>, *Z*_5_ = <0.5776, 0.1990>.

Calculating the score values of these integrated intuitionistic fuzzy information, we can get:

*S*(*Z*_1_) = −0.1480, *S*(*Z*_2_) = 0.3241, *S*(*Z*_3_) = 0.2240, *S*(*Z*_4_) = 0.1464, *S*(*Z*_5_) = 0.3787.

According to these scores and the linear order relation between IFVs, all alternatives can be ranked as the following order: $G_{5}≻G_{2}≻G_{3}≻G_{4}≻G_{1}$.

Using the method proposed in Reference [44] based on $E_{M}^{1.5}$ and $CE_{M}^{1.5}$, we can solve this MADM problem in such a case. The weighting vector can be obtained as: $w={\left(0.19,0.16,0.35,0.30\right)}^{T}$. The ranking order of all alternatives is $G_{5}≻G_{2}≻G_{3}≻G_{4}≻G_{1}$, which is identical to the ranking order obtained by our proposed method. This indicates that the proposed method for solving MADM is reasonable and effective.

**Case** **2.** *There is no information for the attribute weights. Based on the proposed knowledge measure, we can calculate the total knowledge amount of all intuitionistic fuzzy information under one attribute. They are listed as:*

$K_{1}=\sum_{i=1}^{5}K_{1j}=\sum_{i=1}^{5}K_{S}(r_{1j})=2.5663$; $K_{2}=\sum_{i=1}^{5}K_{2j}=\sum_{i=1}^{5}K_{S}(r_{2j})=2.0436$;

$K_{3}=\sum_{i=1}^{5}K_{3j}=\sum_{i=1}^{5}K_{S}(r_{3j})=2.0230$; $K_{4}=\sum_{i=1}^{5}K_{4j}=\sum_{i=1}^{5}K_{S}(r_{4j})=2.0872$.

The attribute weights can be obtained by Equation (19),

$w_{1}=K_{1}/\sum_{i=1}^{4}K_{i}=0.2943$; $w_{2}=K_{2}/\sum_{i=1}^{4}K_{i}=0.2344$; $w_{3}=K_{3}/\sum_{i=1}^{4}K_{i}=0.2320$; $w_{4}=K_{4}/\sum_{i=1}^{4}K_{i}=0.2393$.

Therefore, the weighting vector is $w={\left(0.2943,0.2344,0.2320,0.2393\right)}^{T}$.

Applying the weighting factors and IFWA operator, we can aggregate the evaluation results of an alternative across all attributes. The integrated result for each alternative is:

*Z*_1_ = <0.4259,0.4697>, *Z*_2_ = <0.6459,0.2806>, *Z*_3_ = <0.5561,0.3493>,

*Z*_4_ = <0.5616,0.2740>, *Z*_5_ = <0.5660,0.2064>.

The scores of the intuitionistic fuzzy evaluation information of all alternatives can be obtained as:

*S*(*Z*_1_) = −0.0438, *S*(*Z*_2_) = 0.3562, *S*(*Z*_3_) = 0.2069, *S*(*Z*_4_) = 0.2876, *S*(*Z*_5_) = 0.3596.

All alternatives can be ranked into the following order: $G_{5}≻G_{2}≻G_{4}≻G_{3}≻G_{1}$.

For comparative analysis, we can also solve this decision-making problem based on Xia and Xu’s method based on $E_{M}^{1.5}$ and $CE_{M}^{1.5}$ [44]. The yielded weighting vector is $w={\left(0.2659,0.2486,0.2370,0.2486\right)}^{T}$. The ranked order of five alternatives is $G_{5}≻G_{2}≻G_{3}≻G_{4}≻G_{1}$. We can see that both of our proposed method based on *K_S_* and the method in Reference [44] can take *G*_5_ as the best choice for investment. Even though the order between *G*_3_ and *G*_4_ obtained by our method is different from that obtained by Xia and Xu’ method, this difference has no effect on choosing the best company to invest. Actually, the solution of an MADM problem only concerns the best alternative. The order of other alternatives is beyond the ultimate goal of an MADM problem.

This example demonstrates that the proposed methods for solving MADM problems are competent to getting reasonable results. Compared with Xia and Xu’s method, our proposed optimal model is easier, which will reduce the computation burden. Moreover, our defined knowledge measure is more concise than the cross entropy and entropy measure defied in Reference [44]. Thus, the priority of the new knowledge measure is also verified.

## 6. Conclusions

In this paper, the definition of knowledge measure for AIFSs is addressed. The axiomatic definition of the knowledge measure is first proposed based on intuitive analysis on the knowledge amount. Then we proposed a new knowledge measure for AIFSs. The properties of the new knowledge measure are investigated from a mathematical viewpoint. Numerical examples are applied to illustrate the performance of the new knowledge measure. Comparative analysis on the experimental results indicates that the proposed knowledge measure is effective in discriminating the knowledge amount of different AIFSs. Unlike other existing measures that may lead to unreasonable results, the proposed new knowledge measure is robust in quantifying the knowledge amount of different kinds of AIFSs. Based on the new knowledge measure, we proposed two methods for determining attribute weights in intuitionistic fuzzy MADM problems in the cases that the information on attribute weights is partially known and completely unknown, respectively. Following the determination of attribute weights, we develop a new method to solve the MADM problem. To verify the effectiveness and rationality of the proposed models and the MADM method, we take an MADM problem as an example to make a comparison. The results show that the proposed method is effective enough to obtain reasonable results. Moreover, the proposed method has advantages in reducing computation burden and implementation.

We must point out that the proposed knowledge measure is not the only effective knowledge measure for AIFSs. It is also not the most effective one. In the future, further investigation on the definition of a more effective knowledge measure should be carried out. Moreover, a deeper relation between the knowledge amount and entropy is not investigated in this paper. Although we claim that the membership and non-membership grades influence the knowledge amount, the mechanism behind such an influence is not mined. These are also areas to focus on in future research.

## Figures and Tables

**Table 1:** Comparison results of example 1. (counter-intuitive results are in bold type).

	*E_ZL_*	*E_BB_*	*E_SK_*	*E_VS_*	*E*^2^*_HC_*	*K_SKB_*	*K_N_*	*K_G_*	*K_S_*
*A*_1_ = <*x*,0.5,0.5>	**1**	**0**	**1**	**1**	0.5	0.5	0.8660	0.5000	0.5
*A*_2_ = <*x*,0.25,0.25>	**1**	**0.5**	**1**	**1**	**0.6250**	0.25	**0.4330**	0.2500	0.1038
*A*_3_ = <*x*,0.25,0.5>	0.7500	0.2500	0.6667	0.9387	**0.6250**	0.5417	0.7089	0.5313	0.2945
*A*_4_ = <*x*,0.2,0.3>	0.9	**0.5**	0.8750	0.9855	0.6200	0.3125	**0.4259**	0.3250	0.1106

**Table 2:** Comparison results of example 2 (counter-intuitive results are in bold type).

	*E_ZL_*	*E_BB_*	*E_SK_*	*E_VS_*	*E* ^2^ *_HC_*	*K_SKB_*	*K_N_*	*K_G_*	*K_S_*
*A*_1_ = <*x*,0.7,0.2>	**0.5**	**0.1**	0.3750	0.7878	0.4600	0.7625	0.8185	0.7250	0.5344
*A*_2_ = <*x*,0.5,0.3>	0.8	**0.2**	0.7143	0.9635	0.6200	0.5429	0.7000	0.5200	0.3211
*A*_3_ = <*x*,0.5,0>	**0.5**	0.5	0.5	0.5	**0.5**	**0.5000**	0.5000	0.6250	0.2500
*A*_4_ = <*x*,0.5,0.5>	**1**	**0**	**1**	**1**	**0.5**	**0.5000**	0.8660	0.5000	0.5000
*A*_5_ = <*x*,0.5,0.4>	0.9	**0.1**	0.8333	0.992	0.58	0.5333	0.7810	0.5050	0.3950
*A*_6_ = <*x*,0.6,0.2>	0.6	**0.2**	0.5	0.849	0.56	0.6500	0.7211	0.6400	0.3919
*A*_7_ = <*x*,0.4,0.4>	**1**	**0.2**	**1**	**1**	0.64	0.4000	0.6928	0.4000	0.2948
*A*_8_ = <*x*,1,0>	0	0	0	0	0	1	1	1	1
*A*_9_ = <*x*,0,0>	1	1	1	1	**0**	0	0	0	0

**Table 3:** Comparative results of all AIFSs with respect to *B* (counter-intuitive results are in bold type).

	*E_ZL_*	*E_BB_*	*E_SK_*	*E_VS_*	*E* ^2^ *_HC_*	*K_SKB_*	*K_N_*	*K_G_*	*K_S_*
*A* ^0.5^	**0.4291**	**0.0683**	**0.3518**	0.5640	0.3355	**0.7899**	**0.8680**	**0.7633**	0.6532
*A*	**0.4400**	**0.0800**	**0.4073**	0.5233	0.3280	**0.7563**	**0.8641**	**0.7600**	0.6564
*A* ^2^	0.2160	0.0760	0.1677	0.3369	0.2891	0.8782	0.8950	0.8828	0.7579
*A* ^3^	0.1364	0.0752	0.1101	0.2212	0.2602	0.9074	0.9108	0.9230	0.8157
*A* ^4^	0.1082	0.0800	0.0950	0.1612	0.2397	0.9125	0.9133	0.9337	0.8395
